# Atypical Electrophysiological Findings in a Patient with Acute Motor and Sensory Axonal Neuropathy

**DOI:** 10.3389/fneur.2017.00594

**Published:** 2017-11-08

**Authors:** Viviana Versace, Stefania Campostrini, Frediano Tezzon, Sara Martignago, Markus Kofler, Leopold Saltuari, Luca Sebastianelli, Raffaele Nardone

**Affiliations:** ^1^Department of Neurorehabilitation, Hospital of Vipiteno, Vipiteno, Italy; ^2^Research Unit for Neurorehabilitation South Tyrol, Bolzano, Italy; ^3^Department of Neurology, Franz Tappeiner Hospital, Merano, Italy; ^4^Department of Neurology, State Hospital Hochzirl, Zirl, Austria; ^5^Department of Neurology, Christian Doppler Medical Center, Paracelsus Private Medical University of Salzburg, Salzburg, Austria

**Keywords:** Guillain–Barré syndrome, acute motor and sensory axonal neuropathy, axonal conduction failure, nodo-paranodopathy, anti-ganglioside antibodies

## Abstract

Guillain–Barré syndrome (GBS) is an immune-mediated polyradiculoneuropathy with acute onset and rapid clinical worsening; early diagnosis and immunomodulating therapy can ameliorate the course of disease. During the first days, however, nerve conduction studies (NCSs) are not always conclusive. Here, we describe a 73-year-old man presenting with progressive muscular weakness of the lower limbs, ascending to the upper limbs, accompanied by distal sensory disturbances. Neuroimaging of brain and spine and NCSs were unremarkable; cerebrospinal fluid analysis revealed no albuminocytologic dissociation. Based on typical clinical features, and on positivity for serum GD1b-IgM antibodies, GBS with proximal conduction failure at multiple radicular levels was postulated, and a standard regime of intravenous immunoglobulin was administered. Four weeks later, the patient presented with flaccid tetraparesis, areflexia, and reduction of position sense, tingling paresthesias, and initial respiratory distress. Repeat NCS still revealed almost normal findings, except for the disappearance of right ulnar nerve F-waves. A few days thereafter, the patient developed severe respiratory insufficiency requiring mechanical ventilation for 2 weeks. On day 50, NCS revealed for the first time markedly reduced compound muscle action potentials and sensory nerve action potentials in all tested nerves, without signs of demyelination; needle electromyography documented widespread denervation. The diagnosis of acute motor and sensory axonal neuropathy was made. After 3 months of intensive rehabilitation, the patient regained the ability to walk with little assistance and was discharged home. In conclusion, normal NCS findings up to several weeks do not exclude the diagnosis of GBS. Very proximal axonal conduction failure with late distal axonal degeneration should be taken into consideration, and electrodiagnostic follow-up examinations, even employing unusual techniques, are recommended over several weeks after disease onset.

## Introduction

Guillain–Barré syndrome (GBS) is a rapid-onset polyradiculoneuropathy leading to flaccid tetraparesis and sensory disturbances. According to pathology and electrophysiological features, GBS can be classified into acute inflammatory demyelinating polyneuropathy (AIDP) and acute motor axonal neuropathy (AMAN) or acute motor and sensory axonal neuropathy (AMSAN). It is now well known that “axonal” conduction failure that occurs in AMAN/AMSAN is an immune-mediated disruption of the nodal-paranodal region of the axolemma with consequent failure of saltatory conduction (1–3). The conduction failure may be reversible or may progress to distal axonal degeneration (4).

Here, we report a male patient with flaccid tetraparesis and respiratory distress in whom an acute inflammatory polyneuropathy was suspected despite lack of specific abnormal findings in repeated routine electrophysiological tests for more than 3 weeks.

## Case Report

A 73-year-old man was admitted to the emergency department because of progressive weakness in his lower limbs ascending to the upper limbs, accompanied by tingling sensation in his feet beginning 2 days earlier. He denied fever, infections, or diarrhea during the previous weeks.

He had a history of arterial hypertension, one-vessel coronary artery disease, chronic obliterative arteriopathy of inferior limbs, chronic obstructive pulmonary disease, and chronic nicotine exposure. Vital parameters as well as routine laboratory blood tests were normal.

Neurological examination on admission revealed distally accentuated symmetrical tetraparesis, mainly affecting ankle dorsiflexion (grade 3/5 of Medical Research Council Scale), absent deep tendon reflexes in lower limbs, diminished deep tendon reflexes in upper limbs, and hypesthesia to pinprick and vibration distal to the ankles bilaterally; plantar reflexes were normal; the patient was able to walk with assistance with foot-drop on the right side.

Cerebrospinal fluid (CSF) examination was normal on the same day. Polymerase chain reaction for neurotropic viruses in CSF was negative, as well as analysis of *Borrelia burgdorferi*-specific antibodies in CSF and serum.

On day 2 after admission, a cerebral contrast-enhanced magnetic resonance imaging (MRI) showed non-specific bihemispheric white matter lesions. MRI of the whole spine with gadolinium failed to show any signal alteration of the spinal cord or pathologic contrast enhancement; mild signs of degenerative lumbar spondylosis were present.

**Figure 1:**
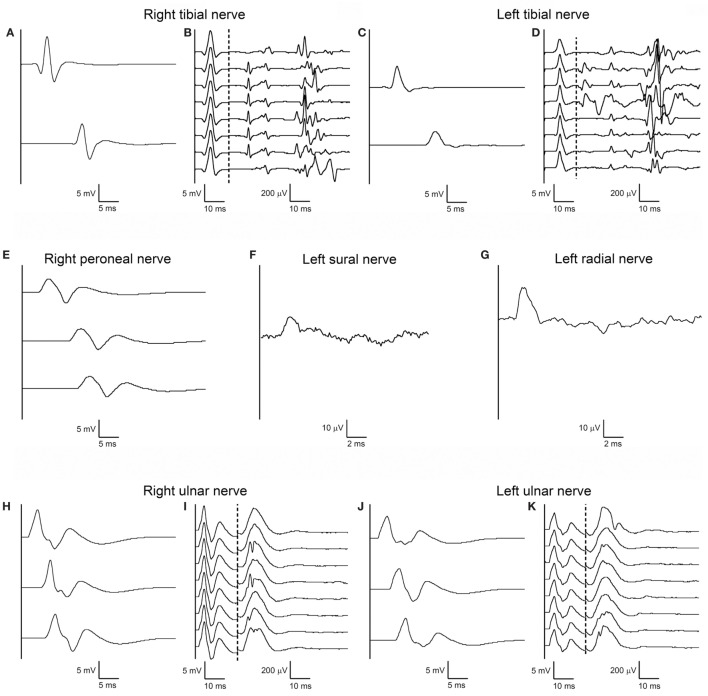
First nerve conduction study (NCS) performed according to standard techniques on day 2 after admission. Right **(A)** and left **(C)** tibial motor NCS from abductor hallucis muscle following stimulation at medial ankle (upper trace) and popliteal fossa (lower trace), and corresponding right **(B)** and left **(D)** F-waves. Right peroneal motor NCS **(E)** from extensor digitorum brevis muscle following stimulation anterior to ankle (upper trace), below fibular head (middle trace), above fibular head (lower trace). Antidromic sensory NCS from left sural nerve **(F)** with recording from posterior ankle following stimulation at posterior-lateral calf, and from left radial nerve **(G)** with recording from snuffbox following stimulation over distal-mid radius. Right **(H)** and left **(J)** ulnar motor NCS from abductor digiti minimi muscle following stimulation at wrist (upper trace), below elbow (middle trace), above elbow (upper trace), and corresponding right **(I)** and left **(K)** F-waves. Right and left median and left peroneal nerves are not shown as their NCSs were also unremarkable. Note that motor and sensory responses of all testes nerves have normal latency, amplitude, and conduction velocity; tibial and ulnar nerve F-waves are normal bilaterally for latency, amplitude, configuration, and persistence; some A-waves are present bilaterally in the tibial nerve F-wave study.

On the same day, nerve conduction studies (NCSs) of upper and lower limbs were performed using standard electrodiagnostic equipment (Viking EDX System, Natus, Middleton, WI, USA). Motor NCSs were obtained from ulnar, median, tibial, and peroneal nerves bilaterally; F-waves were elicited in ulnar and tibial nerves bilaterally, using supramaximal stimuli at wrist and ankle, respectively, at 1 Hz stimulation rate for eight consecutive trials; sensory NCSs were obtained from left sural and left radial nerves. All motor and sensory electroneurographies were normal, including F-waves (which were normal for minimal latency, persistence, amplitude, and morphology), except for the presence of A-waves in tibial nerves bilaterally (Figure 1, right and left median and left peroneal nerves not shown).

On day 3, anti-ganglioside antibody profile was assessed by an immunodot assay, which revealed positivity for GD1b-IgM antibodies.

The patient’s clinical condition worsened, and on day 4 he presented with severe distally accentuated tetraparesis. According to Brighton’s diagnostic criteria the diagnosis of GBS was postulated with a grade 2 of diagnostic certainty (5).

On day 5, a standard regime of intravenous immunoglobulin (IVIg) 0.4 g/kg/day for 5 days was initiated. Despite IVIg therapy, the patient developed respiratory muscle weakness with nocturnal hypercapnia during the third week since admission.

On day 20, repeat CSF examination showed elevated protein and albuminocytologic dissociation (133 mg/dl).

**Figure 2:**
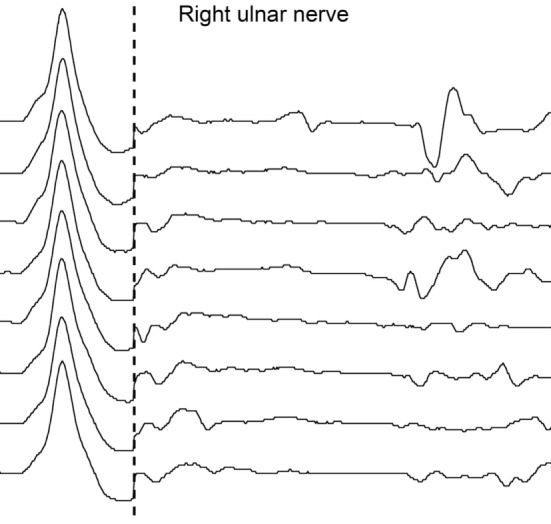
Second nerve conduction study according to standard techniques performed on day 25 after admission. No F-waves are recorded by supramaximal stimulation of right ulnar nerve at wrist; M waves are within normal range.

Five days later, repeat NCS revealed absent F-waves in right ulnar nerve (Figure 2), whereas F-waves in left ulnar and both tibial nerves still showed normal persistence and normal minimal latency; A-waves were still present in tibial nerves; there was no alteration of amplitude, morphology or duration of compound muscle action potentials (CMAPs) or sensory nerve action potentials (SNAPs); motor and sensory conduction velocities were all within the normal range; no conduction blocks were detected. Repetitive stimulation at 3 Hz of ulnar and tibial nerves to rule out neuromuscular transmission failure did not show abnormal amplitude increment or decrement. Needle electromyography (EMG) revealed no spontaneous activity in tibialis anterior bilaterally and left dorsal interosseous; a rapid rate single-unit pattern of few motoneurons discharging at 25–30 Hz was detected during maximal volitional effort.

Four weeks after admission, acute respiratory insufficiency required endotracheal intubation and mechanical ventilation, followed by tracheostomy 15 days later.

The patient’s condition remained stable for the ensuing 2 weeks, so that progressive weaning from ventilation was successfully possible in the seventh week. The patient was then able to breathe spontaneously through the tracheostomy without oxygen supplementation. Repeat neurological examination revealed distally accentuated flaccid tetraparesis, widespread muscle atrophy, absence of deep tendon reflexes, severe reduction of position sense distally and tingling paresthesias in all four limbs, and moderate upper limb ataxia. On day 40 after admission, the patient was successfully decanulated.

Repeat gadolinium-enhanced spinal MRI on the same day did again not show any spinal cord signal changes on T1- and T2-weighted images.

**Figure 3:**
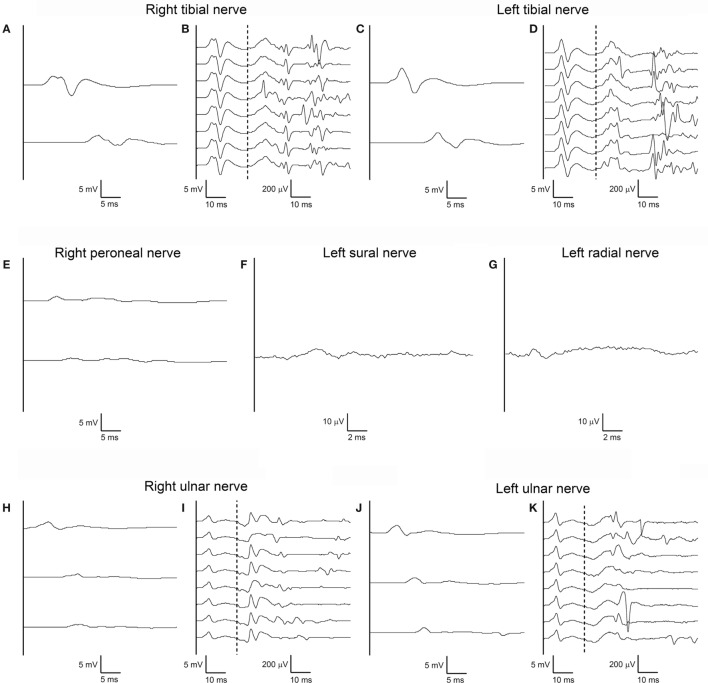
Third nerve conduction study performed according to standard techniques on day 50 after admission. Same nerves, stimulation, and recording conditions as in Figures 1A–K **(A–K)**. Note marked amplitude reduction without temporal dispersion of both compound muscle action potentials and sensory nerve action potentials in all tested nerves with almost normal conduction velocities. Tibial nerve F-waves remain present and normal bilaterally, with some A-waves still present. Right ulnar nerve F-waves are now absent, with some A-waves present; left ulnar F-waves show reduced amplitude.

A repeat electrodiagnostic examination on day 50 revealed only slightly reduced conduction velocities, but then for the first time marked amplitude reduction without temporal dispersion of both CMAPs and SNAPs in all tested nerves; tibial nerve F-waves were still present bilaterally, with normal persistence, morphology and minimal latency; right ulnar nerve F-waves were still absent, and some A-waves were detected, left ulnar F-waves showed reduced amplitude (Figure 3). Needle EMG documented widespread spontaneous activity, subacute neurogenic restructuring of motor unit action potentials, and a reduced interference pattern.

Within 4 months of intensive neurorehabilitation, the patient regained the ability to walk with a walker and to perform activities of daily living with little assistance. He was discharged home 6 months after disease onset.

## Discussion

We report a patient with an AMSAN variant of GBS in whom initial diagnosis was made solely on the basis of typical clinical features and course and positivity of anti-ganglioside antibodies (GD1b-IgM) in serum, after ruling out extensive longitudinal myelitis on repeat MRI. Initial motor and sensory NCSs on days 2 and 25 after hospitalization were unremarkable, and first electrodiagnostic findings concurring with AMSAN occurred later than 4 weeks after disease onset.

The unique early abnormal finding was the presence of A-waves in recordings of late responses in tibial nerves bilaterally (Figure 1) and the absence of F-waves in the right ulnar nerve, but only in the second examination (Figure 2).

However, detection of A-waves in both tibial nerves is in fact a non-specific finding, being frequently observed in the lower extremities in elderly healthy subjects (6). The disappearance of right ulnar nerve F-waves as an isolated abnormal finding in the second NCS on day 25 was in stark contrast to the patient’s serious clinical condition, and as such not sufficient to solve the diagnostic problem.

Severe flaccid (“peripheral-type”) paresis in the presence of almost normal NCSs in the first two examinations up to 4 weeks from disease onset is certainly exceptional and concurs with proximal conduction failure at multiple segmental levels. At that time, there was also no evidence of axonal degeneration, based on normal CMAP and SNAP amplitudes. A recent study in a cohort of GBS patients reported normal NCS in 37% within the first 4 days after symptom onset (7). Yet, normal NCS including normal F-waves in multiple nerves some 4 weeks after disease onset in a patient presenting with flaccid tetraparesis and respiratory distress remains difficult to explain. We assume that a substantial number of motor axons in the anterior roots must have been afflicted by conduction failure due to the immune attack. Perhaps few unblocked axons may have been sufficient to generate normal late responses. In fact, only about 5–10% of alpha-motoneurons generate F-waves in response to an electrical stimulus (8).

It usually takes 2–3 weeks following a proximal axonal nerve lesion for the ongoing Wallerian degeneration to reach distal sites, and to manifest as pathological spontaneous activity on EMG (9). Absence of electrophysiological signs of distal axonal degeneration some 4 weeks after disease onset remains unusual. A possible explanation could be a variable time of onset of distal axonal degeneration occurring in an immune-mediated proximal conduction failure at the nodes of Ranvier as opposed to the time course after nerve transection, particularly if the immune attack is directed only against very proximal sites (10, 11). Alternatively, it could be explained by the occurrence, in some proportion, of early reversible conduction failure (RCF) in the nodal region of nerve roots. The overall incidence of RCF in serially tested nerves of patients with axonal GBS variants has recently been estimated 42.8% (12). Several human and experimental studies suggest that the proportion between RCF with rapid restoration of the nodal-paranodal region, and persistent conduction failure with secondary distal axonal degeneration determine the amount of clinical recovery (4). The very favorable outcome of the herewith reported patient could be at least partly due to proximal RCF, together with efficient distal reinnervation through compensatory collateral sprouting as documented by enlarged and polyphasic MUAPs in the last EMG examination.

In two histopathological studies, very mild findings (in terms of axonal loss or fiber degeneration) were reported in some AMAN patients despite severe paralysis (13, 14).

Kuwabara et al. reported a retrospective series of 12 patients with GBS and reduced F-waves (persistence <20% or total absence) as an isolated electrophysiological abnormality in two or more nerves. In their follow-up study, half of patients presented with recovery of F-waves within 2 weeks, without developing distal axonal degeneration (CMAPs remained within normal range in all consecutive examinations up to 3 months); these patients also recovered from their clinical deficits within few months. In the other half of patients, F-waves remained absent, distal CMAP amplitudes declined about 1 week after the first electrodiagnostic examination, probably because of additional lesions in distal motor nerve segments, and their clinical recovery was delayed and incomplete (15).

In the present patient, only in the third electrophysiological examination, which was performed more than 7 weeks after disease onset, and following a 3-week stay in the intensive care unit, NCS revealed significant amplitude reduction of CMAPs and SNAPs in all four limbs, without evidence of demyelination (Figure 3). Needle EMG demonstrated active axonal damage. A possible role of critical illness polyneuropathy in triggering or accelerating axonal degeneration needs to be taken into consideration, even if the occurrence of critical illness polyneuropathy in a patient with a history of mechanical ventilation, but no signs of systemic inflammatory response syndrome, sepsis, or multiorgan failure are unlikely (16).

As NCSs were not diagnostic for a long time in the course of this patient’s disease, additional laboratory parameters had to be taken into consideration to establish the diagnosis. Anti-ganglioside antibodies GD1b of the IgM and IgG class have previously been described in association with AIDP (17), in GBS/Miller Fischer overlap syndrome patients (18) as well as in acute and chronic idiopathic sensory ataxic neuropathy (ASAN) (19, 20). Thus, based on the presence of these antibodies in the patient’s serum, the diagnosis of AMSAN was considered, despite negative electrodiagnostic features at that time.

The reversible or persistent immune-mediated disruption of the nodes of Ranvier (“nodo-paranodopathy”) through antibodies against gangliosides GM1 or GD1a (in AMAN and AMSAN forms) or GD1b (in ASAN forms) is recognized as underlying pathological mechanism in acute axonal polyneuropathies and determines the muscle weakness (21); the immune attack may occur along the entire peripheral nerve, but the axolemma of the nerve roots is more susceptible because of the greater permeability of the local blood–nerve barrier (22).

The occurrence of extensive Wallerian-like degeneration of large myelinated motor and sensory fibers, as well as pathological nodal lengthening, in the ventral and dorsal spinal roots of AMSAN patients was reported in pathological studies (23–25) being the exclusive pathological marker in two of these patients, who died very early (7 days) after disease onset (25).

An immune-mediated nodal disruption was also documented in dorsal roots of Gd1b immunized ASAN rabbit models (21).

## Conclusion

Limitations of this study—as becoming evident on retrospective—are threefold. (a) If the intervals of electrodiagnostic reexaminations would have been reduced, the first conclusive signs of axonal degeneration, i.e., amplitude reduction of CMAPs and SNAPs, as well as spontaneous activity on needle EMG, might perhaps have been detected earlier, between day 25 and day 50 after admission. (b) Attempting to assess conduction in more proximal nerve segments, i.e., proper electrical stimulation at Erb’s point to activate the arm plexus may have allowed for earlier detection of decreased CMAP amplitudes due to proximal-to-distal progression of axonal degeneration in upper limb nerves; furthermore, electrical cervical root stimulation, as well as the triple stimulation technique (26), may have revealed very proximal nodal-paranodal conduction failure early on. (c) Superimposed critical illness polyneuropathy—albeit unlikely—cannot entirely be ruled out in this patient.

At any rate, in selected cases with clinically suspected inflammatory neuropathy, but initially negative electrodiagnostic findings, every attempt should be undertaken, even employing unusual techniques, repeated at short intervals, and for times exceeding the commonly purported 2–3 weeks, to arrive at a conclusive diagnosis.

## Ethics Statement

Ethical approval or patient consent is not required according to national guidelines. *Informed consent*: a written informed consent was obtained from the patient for the publication of this case report.

## Author Contributions

VV performed the acquisition of data, the drafting/revising of the manuscript and accepted responsibility for conduct of research and final approval. SC, FT, and SM performed the acquisition of data and accepted responsibility for conduct of research and final approval. LuS, LeS, MK, and RN performed the drafting/revising of the manuscript and accepted responsibility for conduct of research and final approval.

## Conflict of Interest Statement

The authors declare that the research was conducted in the absence of any commercial or financial relationships that could be construed as a potential conflict of interest.
